# Drought-induced ABA, H_2_O_2_ and JA positively regulate *CmCAD* genes and lignin synthesis in melon stems

**DOI:** 10.1186/s12870-021-02869-y

**Published:** 2021-02-08

**Authors:** Wei Liu, Yun Jiang, Yazhong Jin, Chenghui Wang, Juan Yang, Hongyan Qi

**Affiliations:** 1 Key Laboratory of Protected Horticulture of Education Ministry and Liaoning Province, College of Horticulture, Shenyang Agricultural University, National & Local Joint Engineering Research Center of Northern Horticultural Facilities Design & Application Technology (Liaoning), Shenyang, 110866 Liaoning People’s Republic of China; 2 Vegetable Research Institute, Liaoning Academy of Agricultural Sciences, Shenyang, 110161 Liaoning People’s Republic of China; 3 College of Agriculture, Heilongjiang Bayi Agricultural University, Daqing, 163319 Heilongjiang People’s Republic of China; 4 College of Ecology and Garden Architecture, Dezhou University, Dezhou, 253023 People’s Republic of China

**Keywords:** Oriental melon, Cinnamyl alcohol dehydrogenase, Drought, Lignin, Abscisic acid, Hydrogen peroxide, Jasmonic acid

## Abstract

**Background:**

Cinnamyl alcohol dehydrogenase (CAD) is an important enzyme functions at the last step in lignin monomer synthesis pathway. Our previous work found that drought induced the expressions of *CmCAD* genes and promoted lignin biosynthesis in melon stems.

**Results:**

Here we studied the effects of abscisic acid (ABA), hydrogen peroxide (H_2_O_2_) and jasmonic acid (JA) to *CmCADs* under drought stress. Results discovered that drought-induced ABA, H_2_O_2_ and MeJA were prevented efficiently from increasing in melon stems pretreated with fluridone (Flu, ABA inhibitor), imidazole (Imi, H_2_O_2_ scavenger) and ibuprofen (Ibu, JA inhibitor). ABA and H_2_O_2_ are involved in the positive regulations to *CmCAD1*, *2*, *3*, and *5*, and JA is involved in the positive regulations to *CmCAD2*, *3*, and *5*. According to the expression profiles of lignin biosynthesis genes, ABA, H_2_O_2_ and MeJA all showed positive regulations to *CmPAL2-like*, *CmPOD1-like*, *CmPOD2-like* and *CmLAC4-like*. In addition, positive regulations were also observed with ABA to *CmPAL1-like*, *CmC4H* and *CmCOMT*, with H_2_O_2_ to *CmPAL1-like*, *CmC4H*, *CmCCR* and *CmLAC17-like*, and with JA to *CmCCR*, *CmCOMT*, *CmLAC11-like* and *CmLAC17-like*. As expected, the signal molecules positively regulated CAD activity and lignin biosynthesis under drought stress. Promoter::GUS assays not only further confirmed the regulations of the signal molecules to *CmCAD1~3*, but also revealed the important role of *CmCAD3* in lignin synthesis due to the strongest staining of *CmCAD3 promoter::GUS*.

**Conclusions:**

*CmCADs* but *CmCAD4* are positively regulated by ABA, H_2_O_2_ and JA under drought stress and participate in lignin synthesis.

**Supplementary Information:**

The online version contains supplementary material available at 10.1186/s12870-021-02869-y.

## Background

Lignin is the second rich substance in plants [1] and plays important roles in sap transport and water barrier in addition to the basal function of support [2]. In lignin monomer synthesis pathway, cinnamyl alcohol dehydrogenase (CAD) is an important enzyme which functions in the last step responsible for the transformation between cinnamyl aldehydes and cinnamyl alcohols. Lignin synthesis is not only complied with regular growth, but also can be regulated by biotic and abiotic stresses. As one frequently happened abiotic stress, drought negatively affects plant growth but promotes lignification process. Some research showed that lignifying enzymes (cinnamoyl CoA reductase (CCR), CAD, kinds of peroxidases) were induced and lignin synthesis was promoted in shoots under drought stress [3–6]. While in roots, lignin biosynthesis was suppressed at the beginning and induced in the late period when suffering drought stress [7–9]. The positive promotion of drought on lignification may confer to plants drought tolerance in aspects of water transport [10] and water loss [11].

Drought-affected lignification is mainly regulated by stress-induced signal molecules, such as abscisic acid (ABA), hydrogen peroxide (H_2_O_2_), jasmonic acid (JA), salicylic acid (SA), and etc. Previous studies reported that JA can induce *CAD* gene expression [12] and promote lignin deposition together with reactive oxygen species (ROS) [13, 14]. H_2_O_2_ can control lignification not only in shoots with light [15], but also in roots without light [16]. While the regulation of ABA to lignification still remains controversial, Mohr and Cahill (2007) [17] reported that ABA inhibited lignin synthesis and SA accumulation in Arabidopsis leaves, however studies also found that ABA increased expressions of lignin biosynthesis genes (phenylalanine ammonia (*PAL)*, cinnamate 4-hydroxylase (*C4H*), 4-coumarate-CoA ligase (*4CL5*), *CAD*, *CCR*, ferulate 5-hydroxylase (*F5H1*)) and activities of lignifying enzymes (PAL, peroxidase (POD)), thus promoted the accumulation of lignin [18–20]. In addition, positive correlations were also observed between ABA, JA, SA and lignification in mature and senescence tissues [21, 22].

As an important enzyme in lignin monomer synthesis pathway, activity of CAD enzyme and expression levels of *CAD* genes were promoted by drought and signal molecules, resulting in lignin deposition [5, 16, 23]. *CAD* genes usually present as a gene family in different species and each member may function distinctly or redundantly from each other [24, 25]. Our team discovered five *CmCAD* genes in melon genome database (http://melonomics.cragenomica.es/) [26] and found they can be significantly up-regulated by drought stress in melon seedlings [27]. However, it remains unclear which signal molecule is responsible for regulating *CmCADs* under drought stress.

In this study, we investigated the regulations of ABA, H_2_O_2_ and JA to the five *CmCAD* genes, as well as some lignin biosynthesis genes, and lignin deposition through using their corresponding inhibitor or scavenger. Then promoter::GUS assays were performed to investigate the regulations of the signal molecules to *CmCAD1*, *2*, and *3*, which members were chosen according to the phylogenetic analysis [26] and the expression profiles under drought stress [27], as well as the expression patterns in melon seedlings (unpublished data). The results revealed important roles of ABA, H_2_O_2_ and JA in regulating *CmCAD1~3*, *5* and lignin biosynthesis genes, thus in regulating lignin biosynthesis under drought stress.

## Results

### Drought-induced ABA, H_2_O_2_ and MeJA could be inhibited by the corresponding inhibitor or scavenger

**Fig. 1:**
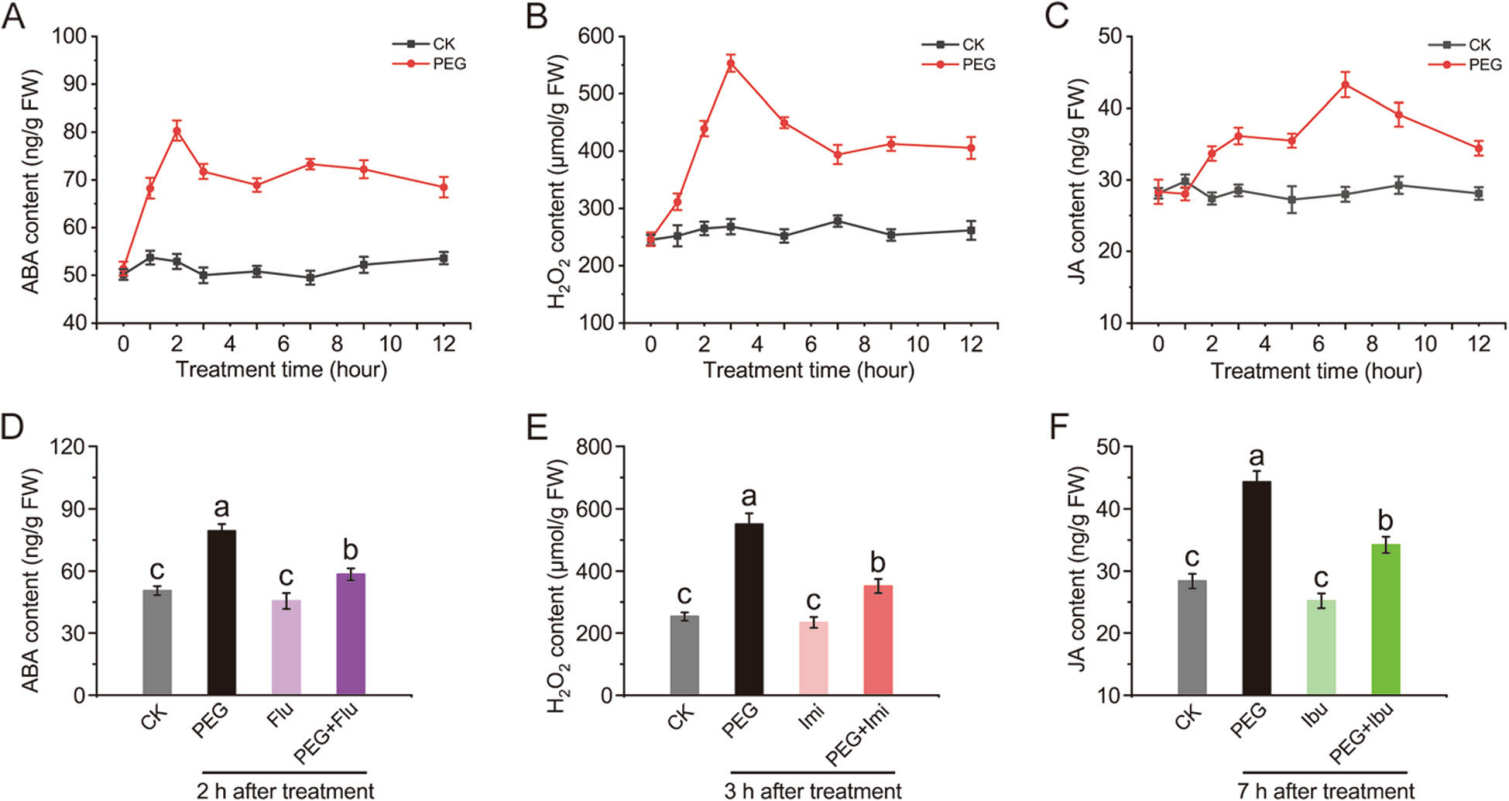
Contents of ABA (**a**, **d**), H_2_O_2_ (**b**, **e**) and JA (**c**, **f**) in stems of melon seedlings. **a**-**c**, changes of ABA, H_2_O_2_ and JA contents under 8% PEG6000 treatment. **d**-**f**, contents of ABA, H_2_O_2_ and JA under PEG and/or inhibitor or scavenger treatments with respect to ABA, H_2_O_2_ and JA at peak-value time point according to **a**-**c**. Values are the means ± SE of three independent experiments. Different lowercase letters indicate significant differences from control using Duncan’s Multiple Range Test at *P*< 0.05

ABA and H_2_O_2_ are frequently reported as important signal molecules in regulating downstream gene expression and metabolism under drought stress [6, 28]. While, it remains controversial of the role of JA under drought stress [29]. To investigate the changes of ABA, H_2_O_2_ and JA in melon stems under drought stress, contents of ABA, H_2_O_2_ and JA were determined and found the signal molecules were all induced by PEG treatment (Fig. 1a-c). ABA and H_2_O_2_ responded to drought fast and reached peak value at 2 h (1.6 fold) and 3 h (2.26 fold), respectively, while JA reached peak value at 7 h (1.54 fold) after PEG treatment. Though decreases were observed after reaching peak values, contents of the signal molecules still maintained higher levels than those in control.

Fluridone (Flu), imidazole (Imi) and ibuprofen (Ibu) are used as inhibitor or scavenger in investigating the functions of ABA, H_2_O_2_ and JA in plants [30, 31]. Here, we evaluated the inhibitory efficiency of Flu, Imi and Ibu to ABA, H_2_O_2_ and JA, respectively, in melon stems under drought stress. As shown in Fig. 1d-f, the signal molecules slightly decreased when treated with Flu, Imi or Ibu alone, showing 9.5, 7.5 and 11% decreases compared to those in control, respectively, but were significantly restricted from increasing in PEG-treated melon stems pretreated with Flu, Imi and Ibu, showing 26.4, 36.1 and 22.7% decreases compared to those in PEG-treated samples, respectively. Thus, they can also be used as effective inhibitor or scavenger in melon seedlings for further study.

### *CmCADs* were positively regulated by ABA, H_2_O_2_ and MeJA under drought

**Fig. 2:**
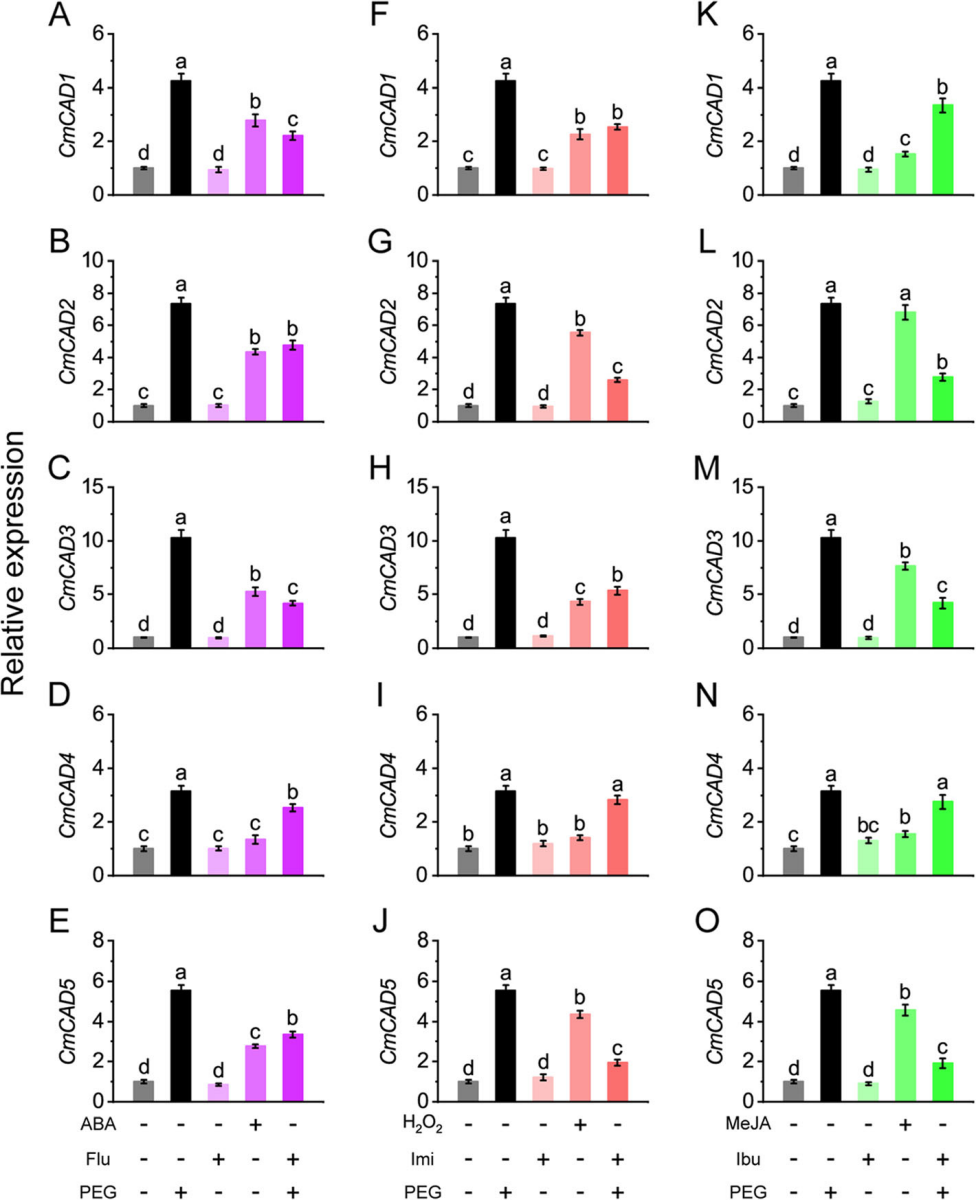
qRT-PCR analysis the relative expressions of *CmCAD* genes in stems of melon seedlings regulated by ABA (**a**-**e**), H_2_O_2_ (**f**-**j**) and MeJA (**k**-**o**). Values are the means ± SE of three independent experiments. Different lowercase letters indicate significant differences from control using Duncan’s Multiple Range Test (*P*< 0.05)

The relationship between drought-induced *CmCADs* [27] and the signal molecules is investigated. As shown in Fig. 2, expression profiles of *CmCAD1~5* increased 4.2, 7.3, 10.3, 3.2, 5.6 fold, respectively, under PEG treatment. ABA treatment significantly up-regulated the expressions of *CmCAD1* (2.8 fold), *2* (4.4 fold), *3* (5.2 fold), and *5* (2.8 fold), and Flu pretreatment significantly inhibited the up-regulations of them in PEG-treated melon seedlings (2.2, 4.8, 4.2, 3.4 fold, respectively). H_2_O_2_ treatment also significantly up-regulated the expressions of *CmCAD1* (2.3 fold), *2* (5.5 fold), *3* (4.3 fold), and *5* (4.4 fold), and Imi pretreatment severely inhibited the up-regulations of them in PEG-treated melon seedlings (2.5, 2.6, 5.3, 2 fold, respectively). MeJA treatment significantly up-regulated the expressions of *CmCAD2* (6.8 fold), *3* (7.6 fold), and *5* (4.6 fold), and Ibu pretreatment strongly inhibited the up-regulations of them in PEG-treated melon seedlings (2.8, 4.2, 1.9 fold, respectively). Notably, MeJA exhibited stronger regulations to *CmCAD2* and *3* than ABA and H_2_O_2_ did. *CmCAD4* was slightly regulated by the signal molecules. Treated with each inhibitor or scavenger alone had no or little effect on *CmCADs* expressions.

### Lignin biosynthesis genes were diversely regulated by ABA, H_2_O_2_ and MeJA under drought

**Fig. 3:**
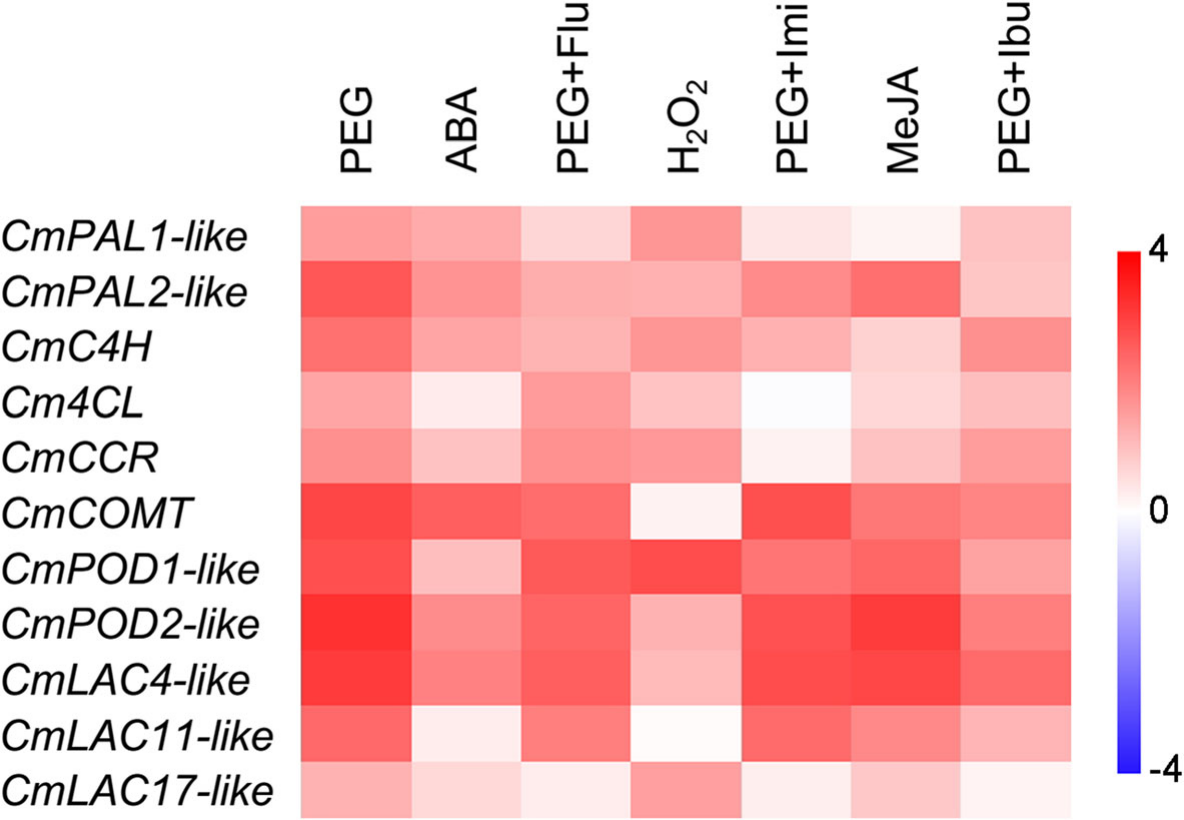
Heat map depicting expression fold changes of lignin biosynthesis genes in lignin monomer synthesis pathway. Data presented as Log_2_[abundance in sample/abundance in control]. Data are collected from biological replicates (*n* = 3)

Thereafter, we also investigated the expression profiles of some lignin biosynthesis genes in the lignin monomer synthesis pathway as shown in Fig. 3. *CmC4H*, *Cm4CL*, *CmCCR* and caffeic acid *O*-methyltransferase (*CmCOMT*) were selected according to Jing et al. (2018) [32]. *CmPAL1*-*like*, *CmPAL2*-*like*, *CmPOD1*-*like*, *CmPOD2*-*like*, laccase (*CmLAC4*-*like*, *CmLAC11*-*like* and *CmLAC17*-*like*) genes are highly homologous to the corresponding genes identified in lignin synthesis in Arabidopsis [33–35].

The lignin biosynthesis genes were all highly up-regulated in expressions under drought stress. Among the genes, *CmPAL1-like*, *CmPAL2-like*, *CmC4H*, *CmCOMT*, *CmPOD1-like*, *CmPOD2-like* and *CmLAC4-like* were induced by ABA treatment and inhibited from increases under PEG+Flu treatment, indicating ABA signalling involves in the regulation of these genes. Similarly, *CmPAL1-like*, *CmPAL2-like*, *CmC4H*, *CmCCR*, *CmPOD1-like*, *CmPOD2-like*, *CmLAC4-like* and *CmLAC17-like* were up-regulated by H_2_O_2_ treatment and restricted from inductions under PEG+Imi treatment, suggesting the positive regulation of H_2_O_2_ to these genes. The lignin biosynthesis genes except for *CmPAL1-like*, *CmC4H* and *Cm4CL* were induced by MeJA treatment and inhibited by PEG+Ibu treatment, implying JA signalling involves in the regulation of these genes. *Cm4CL* studied here was only slightly regulated by H_2_O_2_.

### CAD activity was positively regulated by ABA, H_2_O_2_ and MeJA under drought

**Fig. 4:**
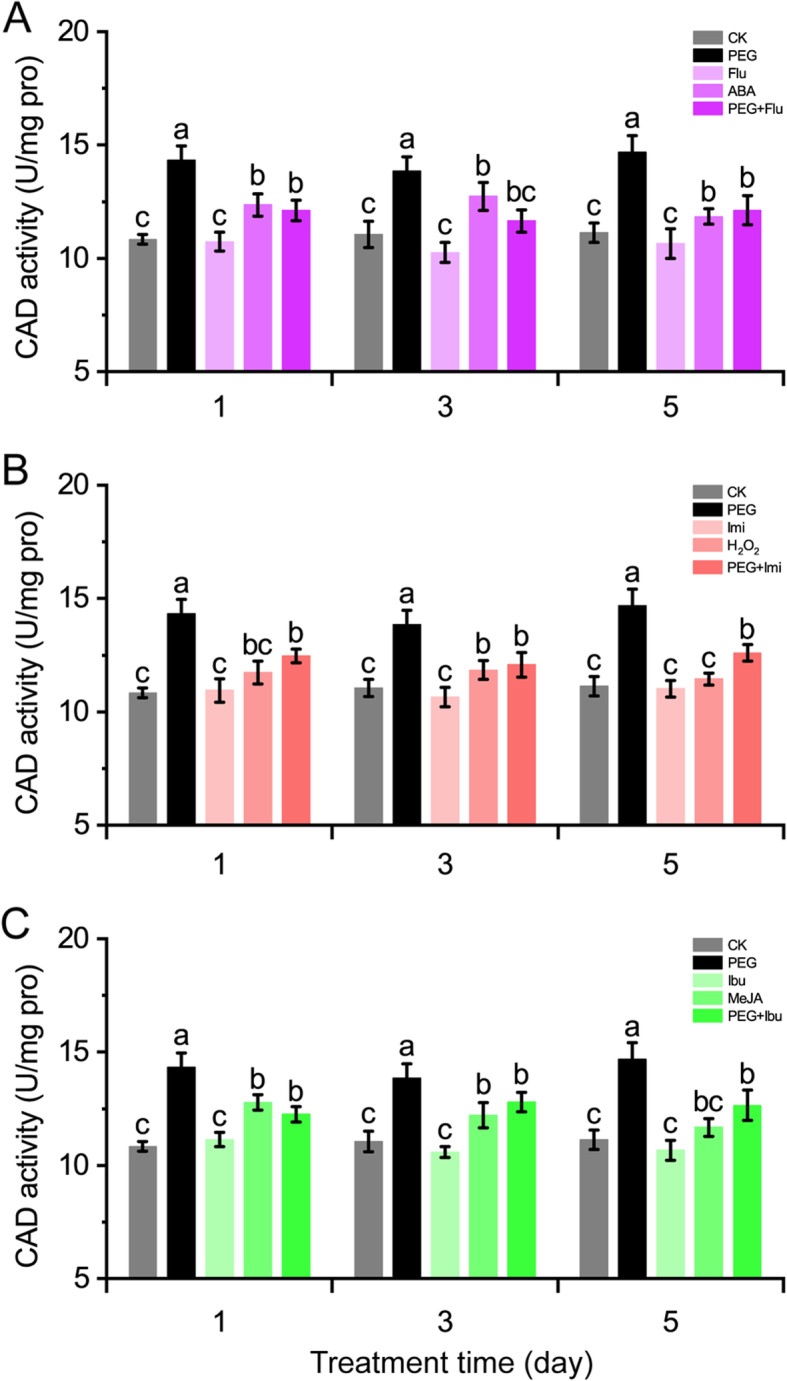
Effects of pretreated with ABA inhibitor fluridone (**a**), H_2_O_2_ scavenger imidazol (**b**) and MeJA inhibitor ibuprofen (**c**) on CAD activity in stems of melon seedlings exposed to PEG. Values are the means ± SE of three independent experiments. Different lowercase letters indicate significant differences from control using Duncan’s Multiple Range Test (*P*< 0.05)

Since ABA, H_2_O_2_ and MeJA play positive roles in regulating the *CmCADs* under drought stress, it is necessary to investigate the effects of the signal molecules on CAD activity. Under various treatments, CAD activity was actively induced under PEG treatment and not affected by each inhibitor or scavenger treatment. Signal molecule treatments promoted CAD activity from increasing in melon stems and inhibitor or scavenger pretreatments inhibited CAD activity from increasing in PEG-treated melon stems (Fig. 4). Among the signal molecules, ABA induced CAD activity to significant levels (increased 6.4–15.2%) (Fig. 4a), and MeJA promoted CAD activity from increasing about 4.9–17.9% (Fig. 4c), while H_2_O_2_ showed slight induction to CAD activity which only reached significant level (7%) at 3 day after treatment (Fig. 4b). Pretreatments of inhibitor or scavenger all strongly suppressed the up-regulation of CAD activity in PEG-treated melon stems. These results suggest that the signal molecules probably involves in the up-regulation of CAD activity by PEG treatment.

### Lignin biosynthesis was positively regulated by ABA, H_2_O_2_ and MeJA under drought

**Fig. 5:**
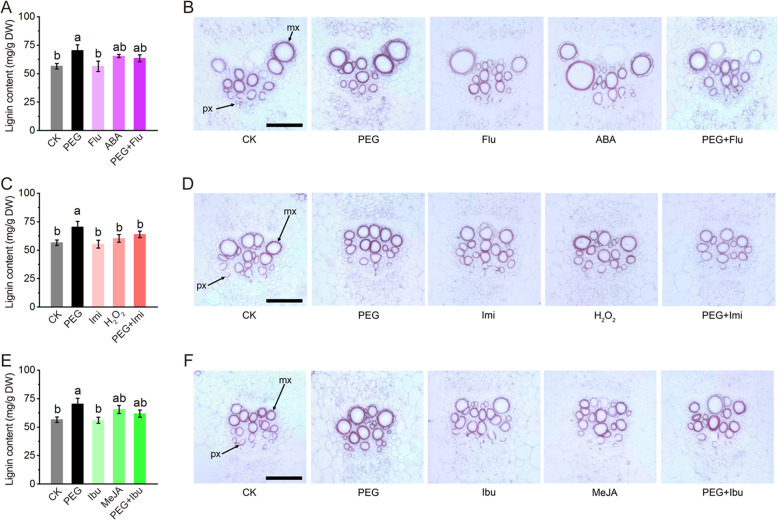
Lignin deposition regulated by ABA, H_2_O_2_ and JA under PEG treatment. **a**, **c**, **e**, Effects of ABA, H_2_O_2_, JA and their corresponding inhibitor or scavenger on lignin contents under PEG treatments. **b**, **d**, **f**, histochemical staining of xylem tissue from the 3rd internode of stems from melon seedlings with phloroglucinol-HCl for lignin observation. mx: metaxylem; px: protoxylem. Different lowercase letters indicate significant differences from control using Duncan’s Multiple Range Test (*P*< 0.05). Bars=50 μm

Further, assays of lignin content and histochemical staining were performed. Results as shown in Fig. 5, lignin biosynthesis in stems was significantly promoted by PEG treatment and inhibited by inhibitor or scavenger pretreatments. Though not to significant levels, the signal molecule treatments showed slight promotions on lignin biosynthesis and deposition in similar patterns (Fig. 5a, c, e). Histochemical staining with phloroglucinol-HCl is a method commonly used for direct lignin observation. Consistent with lignin content detection, xylem tissues in vascular bundles exhibited the strongest staining under PEG treatment, the modest staining under treatments of the signal molecules or pretreatments of inhibitor or scavenger, and the control-like staining under treatments of inhibitor or scavenger (Fig. 5b, d, f). These results suggest that the signal molecules play positive roles in lignin biosynthesis under drought stress.

### Promoters of *CmCAD1*, *2*, and *3* were positively induced by ABA, H_2_O_2_ and MeJA

**Fig. 6:**
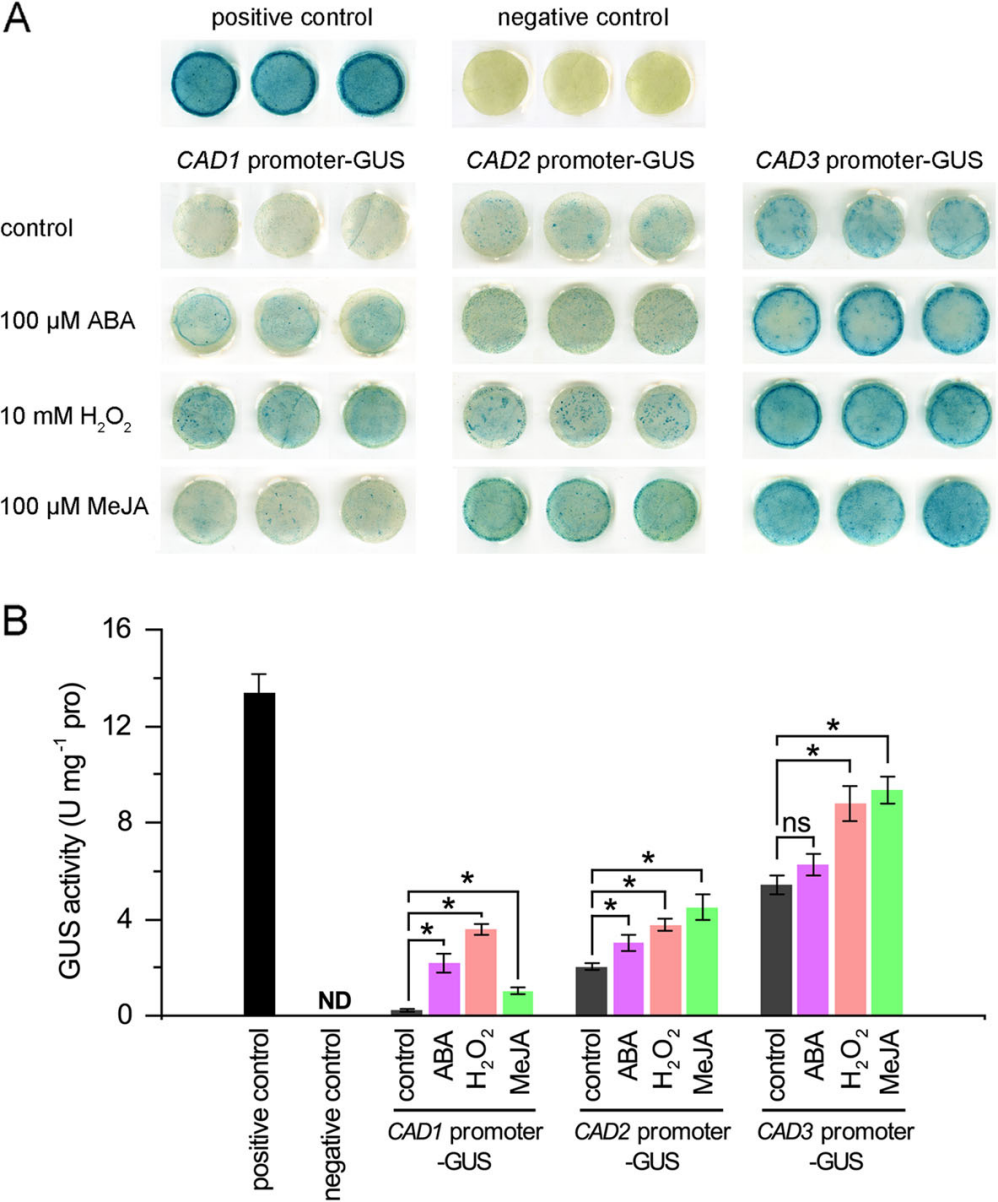
Histochemical staining (**a**) and activity detection (**b**) of GUS driven by promoters with respect to *CmCAD1*–*3* treated with ABA, H_2_O_2_ and MeJA. Different lowercase letters indicate significant differences from control using Duncan’s Multiple Range Test (*P*< 0.05). ND: not detected; ns: no significance

To further study the response of *CmCADs* to the three signal molecules, we constructed promoter::GUS vectors of *CmCAD1 pro::GUS*, *CmCAD2 pro::GUS* and *CmCAD3 pro::GUS* and carried out GUS assays in tobacco leaves treated with ABA (100 μM), H_2_O_2_ (10 mM) or MeJA (100 μM). As Fig. 6a revealed, positive control showed the strongest GUS staining driven by 35S promoter and negative control exhibited no GUS staining without promoter. Under control, *CAD1 pro::GUS* had the slightest GUS staining, *CAD2 pro::GUS* the modest, and *CAD3 pro::GUS* the strongest which is consistent with its highest expression pattern in melon seedlings. Under signal molecule treatments, *CmCAD1 pro::GUS* was slightly induced by MeJA, but was strongly induced by ABA and H_2_O_2_. While *CmCAD2 pro::GUS* and *CmCAD3 pro::GUS* exhibited similar staining patterns, they were both slightly induced by ABA and strongly induced by H_2_O_2_ and MeJA. GUS activity assay, Fig. 6b, obtained similar results with those of histochemical staining. GUS activity driven by *CmCAD1* promoter increased 9.7, 15.9 and 4.6 fold in response to ABA, H_2_O_2_ and MeJA, respectively. GUS activity driven by *CmCAD2* promoter observed 1.5, 1.9 and 2.2 fold increases in response to ABA, H_2_O_2_ and MeJA, respectively. And 1.2, 1.6 and 1.7 fold increases of GUS activity were observed driven by *CmCAD3* promoter in response to ABA, H_2_O_2_ and MeJA, respectively. These results demonstrate that the signal molecules play important roles in regulating *CmCAD1*, *2*, and *3*.

## Discussion

Jin et al. (2014) [26] analyzed the promoters of the five *CmCAD* genes and discovered elements responsible for ABA, H_2_O_2_, JA, stresses and etc. In plants, ABA, H_2_O_2_ and JA are very important signal molecules responsible for plant growth regulation under either normal condition or stressful conditions [36–38]. Drought, as a frequently encountered abiotic stress, usually induce ABA, H_2_O_2_ and JA from highly increasing [6, 39], however, these signal molecules regulate lignification diversely in different species [14, 17, 20, 40]. Thus it becomes necessary to reveal the regulating patterns of these signal molecules to *CmCAD* genes as well as lignification in melon stems under drought stress for adding an understanding.

First of all, we confirmed that ABA, H_2_O_2_ and JA all could be induced significantly by drought stress and reached peak values differentially after PEG treatment, and then they were efficiently restricted from reaching each peak value pretreated with inhibitor or scavenger, implying their signalling to downstream can be weakened efficiently. While, treated with inhibitor or scavenger alone had little effect on the signal molecules as well as thereafter indexes, which is similar with the results observed by Hu et al. (2013), Kojo et al. (2006) and Shan and Liang (2010) [30, 41, 42]. The inhibitor or scavenger exhibited little effect on the signal molecules when treated with each of them alone, but strongly inhibited the singal molecules from increasing under PEG+inhibitor or +scavenger treatments, which can be explained that they do nothing with the existed signal molecules but strongly inhibit the synthesis and accumulation of the signal molecules induced by PEG treatment.

Application of inhibitor or scavenger revealed that ABA and H_2_O_2_ exhibited similar positive regulations to *CmCAD1*, *2*, *3*, and *5*. While, JA showed more direct regulations to *CmCAD2* and *3*, as well as *CmCAD5*, and exhibited slight regulation to *CmCAD1*. *CmCAD4* was slightly affected by the signal molecules. This is consistent with previous *CmCADs* promoter analysis [26]. In the 1.5 kb promoter sequences of *CmCADs*, promoters of *CmCAD1*, *2*, and *5* contain TC-rich repeats (cis-acting element involved in defense and stress responsiveness) which may drive the genes responding to H_2_O_2_. Promoters of *CmCAD1*, *2*, *3*, and *5* contain ABRE element (cis-acting element involved in the abscisic acid responsiveness), and we demonstrated these *CmCADs* can be regulated by ABA. *CmCAD3* promoter contains TGACG-motif and CCAAT-box and *CmCAD5* promoter contains CGTCA-motif, all these three elements act as cis-acting regulatory elements involved in the MeJA-responsiveness, which were also confirmed in this study. In addition, some other stress- and signal-responding elements discovered in *CmCAD* promoters ([26], Table S1) may be a reason why certain increases were still observed in PEG-treated melon seedlings pretreated with inhibitor or scavenger. The positive regulating patterns of the signal molecules to *CmCADs* are consistent with previous reports that one *CAD* gene can be regulated by several signal molecules [19, 43] and different *CAD* members in a family may respond to signal molecules and stresses differentially due to the distinct function each member fulfilled [24, 44]. Expression analysis of lignin biosynthesis genes further confirmed the positive regulations of ABA, H_2_O_2_ and JA to lignin biosynthesis under drought stress. Taken together, these results once again demonstrated that drought mediates gene expression depending on various signal molecules [8, 9, 45].

CAD activity and lignin content was drastically induced by drought stress and slightly induced by signal molecule and PEG+inhibitor or +scavenger treatments. Slight inductions to CAD activity and lignin content by exogenous applications of signal molecules may due to the absorption efficiency as well as time-based effects of exogenous applied signal molecules. While the certain increases of CAD activity and lignin content under PEG+inhibitor or +scavenger treatments may due to the inhibitory efficiency of the inhibitors and scavenger, or exist bypass signalling pathways. These suggest a positive correlation between the signal molecules and CAD activity, resulting in lignin accumulation, which is consistent with previous reports [6]. Among the signal molecules, ABA and H_2_O_2_ showed similar regulations to *CmCAD* genes, but ABA and JA showed similar regulations to CAD activity and lignin deposition. However, Mohr and Cahill (2007) [17] presented an inverse result declaring a negative correlation between ABA and lignin, this may due to the reason that JA and SA are more likely responsible for lignin biosynthesis under biotic stresses [46, 47]. Antagonism and cooperation were both observed between ABA and JA under biotic stress [48, 49], which mechanism between ABA and JA interactions is considered existing both dependent and independent ways [38].

JA is also an important drought-response signal [42, 50] and functions in regulating lignin biosynthesis [21, 51]. Our results proved JA functions actively in promoting lignin biosynthesis in drought-stressed melon stems, which is similarly observed in Arabidopsis [40]. Previous studies on H_2_O_2_ also reported positive regulations to lignification showing that increased H_2_O_2_ content corresponded with higher lignin deposition [52] and decreased H_2_O_2_ content correlated with lower lignin deposition [16]. Consistently, our study also observed positive regulations of H_2_O_2_ to *CmCAD* genes and CAD activity, as well as lignification, in a slight degree which may due to the short lifespan of exogenous applied H_2_O_2_.

Earlier, Jin et al. (2014) [26] analyzed and discovered stress and signal response elements in the promoters of *CmCAD* genes, and later, Liu et al. (2018) [27] revealed that drought could induce *CmCADs* expressions and promote lignin deposition. As the signal molecule and inhibitor or scavenger treatments revealed in this study, *CmCADs* are positively regulated by ABA, H_2_O_2_ and JA. To further demonstrate the regulating patterns, promoter::GUS constructs were constructed by replacing 35S promoter with promoters of *CmCAD1*, *2*, and *3*, respectively, in pCAMBIA1381Z vector. These three genes were chosen according to their positive responses to drought stress [27], phylogenetic analysis [26] and their major functions in lignin synthesis [53]. GUS staining of tabacco leaves after signal molecule treatments obtained similar regulating patterns of the signal molecules to the three *CmCAD* genes consistent with expression assay, demonstrating *CmCAD* genes can be regulated by the stress-induced signal molecules similar as Kim et al. (2010) [43] reported. However, *CmCAD1 pro::GUS* showed the lightest GUS staining and activity either without or with signal molecule treatments, while *CmCAD3 pro::GUS* exhibited the strongest staining and activity under all conditions and *CmCAD2 pro::GUS* the modest. These GUS assays further confirmed the regulations of ABA, H_2_O_2_ and JA to *CmCAD1*, *2*, and *3* on the one hand and demonstrated that *CmCAD3* is probably the major lignification member in oriental melon seedlings on the other hand.

## Conclusions

The three signal molecules responded to drought stress strongly, while ABA and H_2_O_2_ in a fast way. Among the signal molecules, ABA and H_2_O_2_ showed similar positive regulation patterns to *CmCAD1*, *2*, *3*, and *5*, and JA positively regulated *CmCAD2*, *3*, and *5*. In addition, these signal molecules also exhibited positive regulations to most of the lignin biosynthesis genes. CAD activity and lignin content are under regulated by the signal molecules, showing positive correlations between lignification and the signal molecules under drought stress. The thereafter promoter::GUS assay provides a further demonstration that the signal molecules function positively in regulating *CmCAD1*, *2*, and *3*.

## Methods

### Plant material and treatments

Oriental melon (*Cucumis melo* var. *makuwa* Makino) cultivar ‘CaiHong7’ (purchased from Qiqihar Vegetable Research Institution, Qiqihar, Heilongjiang Province, China) was taken as experimental material for lignification related analysis and tobacco (*Nicotiana benthamiana*) preserved in our lab (the key laboratory of Key Laboratory of Protected Horticulture of Education Ministry and Liaoning Province, Shenyang Agricultural University, Shenyang, China.) was used for promoter analysis. Seeds were sterilized before sowing. Melon seedlings were cultivated using Yamasaki melon nutrient solution (half strength) [54] which was refreshed every 2 days and tobacco seedlings were cultivated in pots (soil: peat: compost = 1:1:1), both seedlings were cultivated in a growth chamber (25±2 °C, 14 h/10 h light/dark cycle) in our lab.

Melon seedlings with four fully expended leaves were used for treatments. For drought treatment, PEG-6000 was added to the nutrient solution to the final concentration of 8% (w/v); for fluridone (Flu, ABA inhibitor, 25 μM), imidazol (Imi, H_2_O_2_ scavenger, 10 mM) [30], or ibuprofen (Ibu, JA inhibitor, 1 mM) [31] treatment, inhibitor or scavenger were added to the nutrient solution to the intended concentrations; for ABA (100 μM), H_2_O_2_ (10 mM) or MeJA (100 μM) treatment, seedlings were sprayed with each configured solution of ABA, H_2_O_2_ or MeJA; for inhibitor or scavenger combined with drought treatment, seedlings were pretreated with Flu, Imi or Ibu for 12 h and then transferred to the nutrient solution containing 8% PEG-6000. Untreated seedlings were taken as control. Samples were collected after treatments at 0 h, 1 h, 2 h, 3 h, 5 h, 7 h, 12 h, 1 d, 3 d, 5 d for analysis in August 2018 for the first batch and December 2019 for the second batch (voucher No. Cm-20,180,806-001~010 and Cm-20,191,212-001~010).

Six-week-old tobacco seedlings were used for agro-infiltration. The inoculated tobacco seedlings were firstly cultivated at room temperature under dark for 24 h, and then at room temperature under light for another 24 h. After preliminary 48 h cultivation, the tobacco seedlings were then treated with ABA (100 μM), H_2_O_2_ (10 mM) and MeJA (100 μM), respectively. Leaf discs for GUS staining and samples for GUS activity analysis were then collected after 24 h cultivation at 24 °C under 16/8 light/dark condition after treatments in October 2018 for the first batch and January 2020 for the second batch (voucher No. Cm-20,181,013-001 and Cm-20,200,128-001).

Assays of each index contained three biological replicates and each biological replicate included three analytical replicates. The second and third internodes (counted from base to growth point) of treated oriental melon seedlings were collected and used for assays either with fresh or frozen samples. Tobacco leaves for GUS activity assay were collected as frozen samples. Frozen samples were frozen in liquid nitrogen immediately after collection and stored at − 80 °C.

### Measurements of ABA, JA and H_2_O_2_ contents

Contents of ABA and JA were determined using 0.5 g frozen samples according to Li et al. (2011) [55] using ELISA assay kit (made by the China Agricultural University). Specific monoclonal antibodies were used for ABA and JA analysis and standard curve was built for each hormone according to the manufacturer instruction. Contents of ABA and JA were expressed as ng g^− 1^ fresh weight.

Content of H_2_O_2_ was determined using 0.1 g frozen sample following the manufacturer instruction of H_2_O_2_ content detection kit (Cat#A064, Nanjing Jiancheng Bioengineering Istitute, Nanjing, China). H_2_O_2_ content was expressed as mmol g^− 1^ fresh weight (FW) calculated by the absorbance at 405 nm.

### Measurement of lignin content

Lignin contents were measured according to Zhang et al. (2010) [56] as well as we described previously [27, 53]. Extracted lignin solution was measured at 280 nm. Lignin contents were calculated according to a standard curve and expressed as mg g^− 1^ DW.

### Histochemical lignin staining

The phloroglucinol-HCl staining was the same as described in our previous study [27]. While, 10 μm thickness sections were applied for observation.

### Assay of CAD activity

CAD activity is performed the same as we described previously [27]. Coniferyl alcohol was used as substrate and CAD activity is presented as U mg^− 1^ protein. Protein concentration was measured by using BioRad Protein Assay Kit (Code No.T9310A, TaKaRa, Japan) based on the method described by Bradford (1976) [57].

### RNA extraction, cDNA synthesis and qRT-PCR

The processes of RNA extraction, cDNA synthesis and qRT-PCR were executed according to our previous description [27]. The gene sequences of *CmPAL1-like*, *CmPAL2-like*, *CmCAD1~5*, *CmPOD1-like*, *CmPOD2-like*, *CmLAC4-like*, *CmLAC11-like* and *CmLAC17-like* are achieved in Melonomics (https://www.melonomics.net/), sequences of *18 s* rRNA, *CmC4H*, *Cm4CL1* and *CmCOMT* are achieved in NCBI (https://www.ncbi.nlm.nih.gov/) and sequence of *CmCCR* is achieved in PLAZA (https://bioinformatics.psb.ugent.be/plaza/versions/plaza_v4_dicots/). Gene accession numbers and PCR primer sequences are listed in Table S1. Each-gene/*18S* rRNA ratio at 0 h were set to 1. The 2^-ΔΔ*C*t^ method was used to calculate relative expressions of genes.

### Construction of GUS reporter gene vectors and expression in tobacco

The DNA, used for promoter clone, was extracted from untreated melon leaves using Hi-DNAsecure Plant Kit (Cat#DP350–02, Tiangen, Beijing, China). PrimeSTAR® HS (Premix) (Code No.R040A, TaKaRa, Japan) was used for sequence clone. Promoter sequences of *CmCAD1*, *CmCAD2* and *CmCAD3* were firstly cloned using the primers without restriction enzyme cutting site, then added poly-A tails (Cat#RT124, Tiangen, Beijing, China) and ligated into T-vector (pMDTM19(simple), Code No.3271, TaKaRa, Japan) using T4 DNA ligase kit (Cat#RT406, Tiangen, Beijing, China) for sequencing. Correct sequences, which are the same with the sequences downloaded from Melonomics (https://www.melonomics.net/), were secondly cloned using the primers with restriction enzyme cutting sites and ligated into linearized pCAMBIA1381Z by using Double Digest Protocol with Acc I and Hind III restriction enzymes. TaKaRa MiniBEST DNA Fragment Purification Kit Ver.4.0 (Code No.9761, TaKaRa, Japan) was used for cloned sequence purification when necessary. The constructed vectors were introduced into *Agrobacterium tumefaciens* (strain GV3101) competent respectively using freeze-thaw protocol, and the virus was screened and inoculated with LB medium containing 25 mg L^− 1^ rifampicin and 50 mg L^− 1^ kanamycin. After PCR and gel electrophoresis detection, correct bacterial plaques were inoculated in 50 ml LB liquid medium in the presence of rifampicin and kanamycin in erlenmeyer flasks for 14–16 h. Then, bacterial solutions were centrifuged at 4 °C 5000 rpm for 10 min. The harvested *Agrobacterium* pellets were resuspended to the density of 1.0 at 600 nm in infiltration buffer (10 mM MgCl_2_, 10 mM MES, 0.1 mM acetosyringone). After incubation at room temperature under dark condition for 3 h, infiltration cultures were pressure-injected into fully expended leaves of tobacco plants using a 1 ml needleless syringe avoiding veins. Primers used here are listed in Table S2.

### GUS staining

GUS staining in tobacco was assayed according to the method described by Jefferson et al. (1987) [58]. Discs from inoculated tobacco leaves were incubated in GUS staining solution [50 mM phosphate buffer (pH 7.0), 0.5 mM K_3_Fe(CN)_6_, 0.5 mM K_4_Fe(CN)_6_, 0.1% (v/v), Triton X-100, 10 mM EDTA and 0.5 mg/ml X-Gluc (5-bromo-4-chloro-3-indolyl-β-D-glucuronide)] at 37 °C overnight. The stained samples were then removed chlorophyll with 80% (v/v) ethanol and scanned using an Epson scanner (Epson Expression 12000XL).

### GUS activity

GUS activity was referred to the procedures described by Jefferson et al. (1987) [58] and Wang et al. (2019) [59]. Inoculated tobacco leaf (0.2 g each biological replicate sample) was used for GUS activity detection which is calculated as μmol of substrates converted to products per minute and expressed as U mg^− 1^ protein. BioRad Protein Assay Kit was used to determine the protein concentration of enzyme extracts based on the method of coomassie brilliant blue G-250 described by Bradford (1976) [57].

### Statistical analysis

Data were organized using Excel 2013 and analyzed through SPSS 18.0 using Duncan method with *P* = 0.05 to test the significance. Then Origin 8.0 and Photoshop CS4 were both used for graph generation and beautification.

## Supplementary Information

**Additional file 1 Table S1.** Primers used for qRT-PCR analysis.

**Additional file 2 Table S2.** Primers used for promoter clone.

## Data Availability

The data sets are included within the article and its Additional files. Accession numbers of the genes analyzed in this article can be achieved in Table S1.

## Abbreviations

CAD: Cinnamyl alcohol dehydrogenase
ABA: Abscisic acid
H_2_O_2_: Hydrogen peroxide
JA: Jasmonic acid
MeJA: Methyl jasmonic acid
SA: Salicylic acid
ROS: Reactive oxygen species
Flu: Fluridone
Imi: Imidazol
Ibu: Ibuprofen
PEG: Polyethylene glycol
GUS: β-glucuronidase
qRT-PCR: Quantitative real time polymerase chain reaction
C4H: Cinnamate 4-hydroxylase
4CL: 4-coumarate-CoA ligase
CCR: Cinnamoyl-CoA reductase
COMT: Caffeic acid *O*-methyltransferase
PAL: Phenylalanine ammonia lyase
POD: Peroxidase
LAC: Laccase
F5H: Ferulate 5-hydroxylase
