# Supplementary material for: Drought-induced ABA, H2O2 and JA positively regulate CmCAD genes and lignin synthesis in melon stems
Source: BMC Plant Biol. 2021 Feb 8;21:83. doi: 10.1186/s12870-021-02869-y (PMC7871556; doi:10.1186/s12870-021-02869-y)
Supplement: Supplementary file 1 — Additional file 1 Table S1. Primers used for qRT-PCR analysis. [file 12870_2021_2869_MOESM1_ESM.docx]

Table S1 Primers used for qRT-PCR analysis

| Gene | Accession number | Data source | Primer sequence (5’ to 3’) | |
| --- | --- | --- | --- | --- |
| *CmPAL1-like* | MELO3C014229 | Melonomics | *CmPAL1-like*-F | GGGTTGAGGCGAGCAGTAAG |
|  |  |  | *CmPAL1-like*-R | GATTCAGTGCCACGTCCAAA |
| *CmPAL2-like* | MELO3C025786 | Melonomics | *CmPAL2-like*-F | AGAGGGAGTTAAGGCGAGCA |
|  |  |  | *CmPAL2-like*-R | GTGAGGCAATGTGTGGTTGG |
| *CmC4H* | XM_008458031.2 | NCBI | *CmC4H*-F | TGAACTCGACACCGTACTCG |
|  |  |  | *CmC4H*-R | GAACTCTTCCGGGTTCTTCC |
| *Cm4CL1* | XM_008464733.2 | NCBI | *Cm4CL1*-F | GCAGAAATCGCAAAGCAAGC |
|  |  |  | *Cm4CL1*-R | CATAAACAACCATCGTGAGGAGAAT |
| *CmCCR* | CM00060G01120 | PLAZA | *CmCCR1*-F | CAGAGGATCCGAAGAACAGG |
|  |  |  | *CmCCR1*-R | CCGCGATTATGACGTTCTTT |
| *CmCOMT* | XM_008456726.2 | NCBI | *CmCOMT*-F | GGAAACGAGCGTTTATCCAA |
|  |  |  | *CmCOMT*-R | TCCATAGCCAGAATCGTTCC |
| *CmCAD1* | MELO3C019548 | Melonomics | *CmCAD1*-F | GAGACGCAAGAAGTATTG |
|  |  |  | *CmCAD1*-R | ACTCAGGCATCTTACTAC |
| *CmCAD2* | MELO3C018492 | Melonomics | *CmCAD2*-F | CTTACACTTACGAACTCAG |
|  |  |  | *CmCAD2*-R | CAACTTCCATCACTTCAC |
| *CmCAD3* | MELO3C003735 | Melonomics | *CmCAD3*-F | CCACAACACATCAACCAT |
|  |  |  | *CmCAD3*-R | CATCCGCTAATCTTGCTTA |
| *CmCAD4* | MELO3C005809 | Melonomics | *CmCAD4*-F | CATTGTTGTTCACGAGAG |
|  |  |  | *CmCAD4*-R | CCTATCACTCCAAGAGATT |
| *CmCAD5* | MELO3C023272 | Melonomics | *CmCAD5*-F | GCTGTTAAGATTGCTAAGG |
|  |  |  | *CmCAD5*-R | GTAATCCATTGTCTCTGTTG |
| *CmPOD1-like* | MELO3C003275 | Melonomics | *CmPOD1-like*-F | TGGCGATAATGCGCTACAAA |
|  |  |  | *CmPOD1-like*-R | GTGATGGCTCGTTGAAAGTCC |
| *CmPOD2-like* | MELO3C007935 | Melonomics | *CmPOD2-like*-F | GCAACAGAGCTTCTCACAAAGG |
|  |  |  | *CmPOD2-like*-R | TGGGTTCATTTCAGGGTCAA |
| *CmLAC4-like* | MELO3C009189 | Melonomics | *CmLAC4-like*-F | CACCGTTGGGCTAGGAATTA |
|  |  |  | *CmLAC4-like*-R | AGGCCCAGGTCCTGAATAGT |
| *CmLAC11-like* | MELO3C014234 | Melonomics | *CmLAC11-like*-F | CAAGCCAACAAATTGGGACT |
|  |  |  | *CmLAC11-like*-R | GTCATTAAGCGCAGCATTGA |
| *CmLAC17-like* | MELO3C009250 | Melonomics | *CmLAC17-like*-F | TTTACCGTCGGACTTGGAAC |
|  |  |  | *CmLAC17-like*-R | CCGGGAAATCAGTCCTGTAA |
| *18s* rRNA | XM_008443896.2 | NCBI | *18s* rRNA-F | TCTTTCCTCGTTTGCTTG |
|  |  |  | *18s* rRNA-R | ATTCCTAATCCCTGACCC |
