# Supplementary material for: Drought-induced ABA, H2O2 and JA positively regulate CmCAD genes and lignin synthesis in melon stems
Source: BMC Plant Biol. 2021 Feb 8;21:83. doi: 10.1186/s12870-021-02869-y (PMC7871556; doi:10.1186/s12870-021-02869-y)
Supplement: Supplementary file 2 — Additional file 2 Table S2. Primers used for promoter clone. [file 12870_2021_2869_MOESM2_ESM.docx]

Table S2 Primers used for promoter clone

| Primer | Sequence (5’ to 3’) | Usage |
| --- | --- | --- |
| *CmCAD1* promoter-F | AAACAAAATTGGGAGGGAAGAAG | For *CmCAD1* promoter T-clone |
| *CmCAD1* promoter-R | TGTTGTCGTTGTTGTTGTTTTTG |  |
| *CmCAD2* promoter-F | TACCCCTCTTATGGGATGGGA | For *CmCAD2* promoter T-clone |
| *CmCAD2* promoter-R | TATAGAGAGAAAAGAGAAACAG |  |
| *CmCAD3* promoter-F | AAACAAACCACTAAACTCAAC | For *CmCAD3* promoter T-clone |
| *CmCAD3* promoter-R | TGAAATTCAACGAGAGAGAGAG |  |
| *CmCAD1* promoter-F | CCGTCGACAAACAAAATTGGGAGGGAAGAAG | For *CmCAD1* promoter clone with restriction enzyme cutting site |
| *CmCAD1* promoter-R | CCAAGCTTTGTTGTCGTTGTTGTTGTTTTTG |  |
| *CmCAD2* promoter-F | CGTCGACTACCCCTCTTATGGGATGGGA | For *CmCAD2* promoter clone with restriction enzyme cutting site |
| *CmCAD2* promoter-R | CCAAGCTTTATAGAGAGAAAAGAGAAACAG |  |
| *CmCAD3* promoter-F | CGTCGACAAACAAACCACTAAACTCAAC | For *CmCAD3* promoter clone with restriction enzyme cutting site |
| *CmCAD3* promoter-R | CCAAGCTTTGAAATTCAACGAGAGAGAGAG |  |
